# Examining Patient Characteristics in Bladder Cancer Clinical Trials Involving Immunotherapy

**DOI:** 10.3390/jcm14030879

**Published:** 2025-01-29

**Authors:** Izuagie Ikhapoh, Usomine Atairu, Amanda Jane Reich, Charlene M. Mantia, Xiao X. Wei, Rishi Sekar, Timothy N. Clinton, Matthew Mossanen

**Affiliations:** 1 Department of Medical Science, Harvard Medical School, Boston, MA 02115, USA; 2 Department of Medical Microbiology and Immunology, Creighton School of Medicine, Omaha, NE 68178, USA; 3 Corcoran School of the Arts and Design, The George Washington University, Washington, DC 20006, USA; 4 Center for Surgery and Public Health, Brigham and Women’s Hospital, Boston, MA 02120, USA; 5 Lank Center for Genitourinary Oncology, Dana-Farber Cancer Institute, Boston, MA 02115, USA; 6 Department of Urology, University of Michigan, Ann Arbor, MI 48109, USA; rsekar@med.umich.edu; 7 Department of Urology, Brigham and Women Hospital, Jamaica Plain, MA 02130, USA

**Keywords:** immune checkpoint inhibitor, bladder cancer, demographics, race, and comorbidity

## Abstract

**Background/Objectives**: Comprehending the patient composition of bladder cancer (BC) clinical trials is crucial for effectively designing clinical trials and contextualizing the generated science. In this study, we reviewed publicly available data and explored the demographic information of BC studies administering immune checkpoint inhibitors (ICIs). **Methods**: Trial eligibility was irrespective of government or private sponsorship, and trial activation dates were limited to between 2013 and 2023. The main inclusion criteria were use of ICIs and trials reporting endpoints that include progression-free survival (PFS), overall survival (OS), disease-free survival (DFS), and event-free survival (EFS). The key exclusion terms were review articles and meta-analysis. **Results**: We identified a total of 109 clinical trials with an aggregate total of 8936 enrolled patients. Ninety-six percent identified as Caucasian or White European, and 23% identified as female. Further analyses revealed that 65% of the patients were aged 65 years or older. One-third of the trials listed similar comorbidities, such as cardiovascular disease and diabetes, that were exhibited by approximately 30 percent of the patients. **Conclusions**: Our data suggest that recruitment strategies should be mindful of comorbidities that may interfere with ICI treatments. Additionally, our results are consistent with findings from other reviews that indicate that certain patient groups may be under-represented in BC trials.

## 1. Introduction

In 2023, the American Cancer Society [1] reported that the incidence of BC is still increasing. Furthermore, ongoing research continues to underscore current challenges faced by minority groups in accessing advanced clinical therapies, such as immunotherapy [2].

Though clinical trials are crucial for developing effective therapies, current BC studies suffer from under-representation of certain demographic groups. Older adults, women, and racial minorities are consistently under-represented in BC trials testing novel therapeutics [3]. This lack of diversity can lead to a limited understanding of results from BC trials that may not fully capture the disease’s variability across different populations. Consequently, findings from such studies are not generalizable across the broader population affected by BC. To evaluate generalizability, this study is aimed at using publicly available records to explore the demographics of BC clinical trials.

BC has a higher incidence among Caucasians or White Europeans compared to Black or African Americans (BAAs) [4]. According to Sung (2019), the racial distribution for BC has been described as 81% White, 3.8% Black, 8.8% Hispanic, 5.2% Asian, and 1.2% other races [4]. This under-representation can skew trial outcomes, as diverse genetic backgrounds and environmental exposures are not adequately reflected. Without sufficient racial diversity, trial results may not translate to all patient populations. Consequently, several studies have attempted to conduct race- and/or ethnicity-based systematic reviews of the effect of ICIs on cancer [3,5,6]. Though some studies are underpowered, results suggest that minority groups, such as BAAs and Latinos, demonstrate varying responses to ICI treatment that differ from the majority populations [7,8,9].

Several other studies have shown that women with bladder cancer are less often included in clinical trials compared to men [5,10,11,12]. This is concerning because women may experience different disease trajectories and treatment responses [10,11,12]. The lack of female representation can result in an incomplete understanding of how treatments perform in women, which is critical for developing effective, gender-specific treatment approaches. Statistically, BC affects four times as many men as women [13]. Considering these numbers, there may be a discrepancy between the experiences of women in bladder cancer clinical trials compared to real-world evidence regarding female bladder cancer participants that can impact the generalizability of results.

Given that BC patients are often older [13] and present with age-related comorbidities, it is important to evaluate comorbid conditions that may interfere with ICI treatment and contribute to adverse effects. Hence, it is beneficial to conduct targeted demographic studies to understand the population composition of clinical trials. Potential results from population-based studies can enable generalizable treatment strategies that improve health equity of elderly patients.

Though several systematic reviews have been conducted on BC trials, there may be a benefit to better understanding the composition of patients participating in ICI research in BC.

## 2. Materials and Methods

### 2.1. Eligibility Criteria

This systematic review was based on anonymized demographic data reported in randomized controlled trials (RCTs) published in English [6]. The study was exempt from institutional review board approval. We followed PRISMA guidelines underlined by the checklist. Specifically, we included BC trials published between 2013 and 2023 that examined the efficacy of ICIs and reported any of the following endpoints, including progression-free survival (PFS), overall survival (OS), disease-free survival (DFS), and event-free survival (EFS). We excluded trials that did not report demographic information and involved pregnant or lactating women, and we rejected studies that hospitalized patients without consent or deprived people of liberty.

### 2.2. Search

The search strategy for this systematic review was developed to ensure comprehensive coverage of the literature on trials administering ICIs for the treatment of BC. We used a combination of Medical Subject Headings (MeSH) and free-text terms with Boolean operators in PubMed, EMBASE, and Google Scholar to identify the eligible trials. A total of 109 eligible records were obtained. All adjudications for the final list were determined by the three authors listed. All analyses was performed using stringent search rules that yielded consistent results throughout the selection process.

### 2.3. Study Selection, Data Extraction and Quality Assessment

The Newcastle–Ottawa Scale (NOS) was utilized to calculate the quality and consistency of studies included in the systematic review [14]. We used the nine-point scoring system that was based on the NOS to rank quality as characterized by three key criteria: (1) study population, (2) study arm comparability, and (3) endpoints. A thorough process was bolstered by two independent reviewers (II and UA) that initially screened the titles and abstracts to identify studies that met the distinct inclusion criteria. A third reviewer (MM) was consulted to adjudicate disagreements between II and UA when a consensus could not be reached during the initial ranking process. Studies having a score of 7 or above, with 9 being the maximum, were deemed to be of a high quality. Subsequently, full-text articles on eligible trials were retrieved and included in the systematic review. A PRISMA flow diagram was constructed to map out the study selection process.

### 2.4. Data Synthesis

In our data synthesis, we evaluated or estimated the total number of patients across the different ages, sexes, and racial subgroups participating in trials administering ICIs. Estimated patient counts were reported as percentages based on the total number of participants within each subgroup. The estimated mean age of participants across the numerous eligible studies was 57 years (range: 18 to 91). A percent trial and the number of patients with comorbidities was assessed or estimated from the supplemental section. Some eligible trials did not have comorbidity data, and, in the cases of any other missing data, that trial was excluded from the specific analysis.

## 3. Results

### 3.1. Search Results and Characteristics of Trials

**Figure 1:**
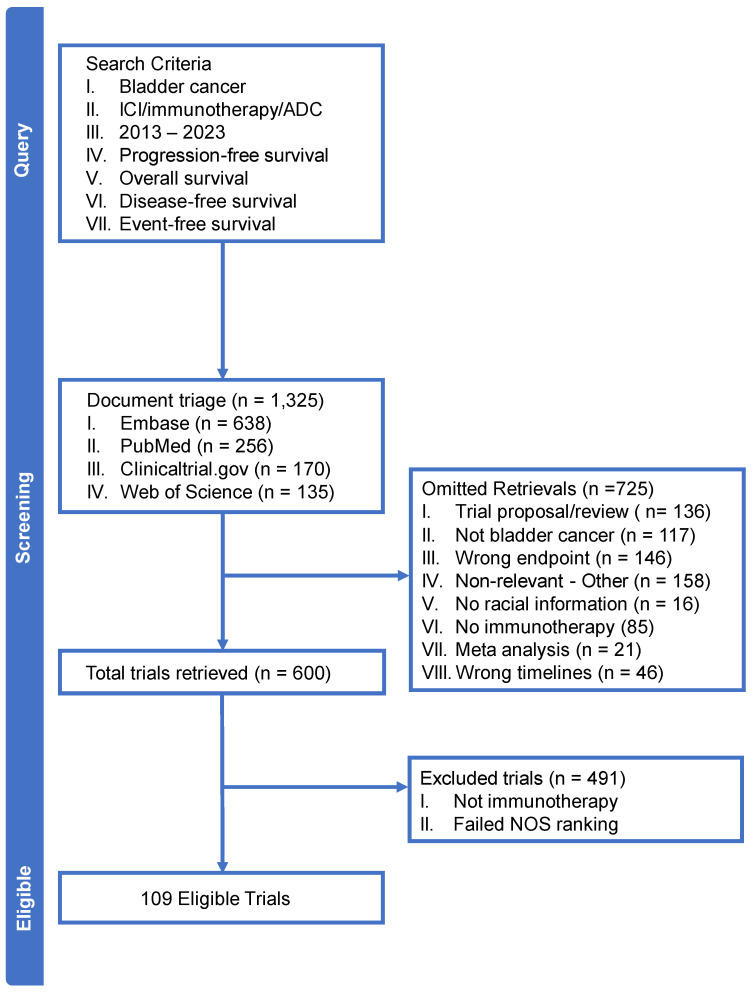
PRISMA diagram illustrating the selection process for eligible studies. Flow chart illustrating the study selection process for this systematic review. The initial search across databases yielded 1325 records. After duplicate removal, 1216 records were screened-out, leading to 109 full-text articles being accepted based on the eligibility criteria included.

Our review of electronic databases yielded 1325 initial records extracted from MEDLINE, Embase, and Web of Science. Out of the initial records, 109 studies investigating ICIs were deemed eligible for analysis. The process for article retrieval is illustrated in Figure 1. The primary reasons for exclusion included trial duplicates, studies unrelated to bladder cancer, an absence of race data, incorrect endpoints, and studies that failed the NOS ranking.

Eligible studies also included multi-armed randomized control trials, where ICIs were used in the experimental arm and chemotherapy was used in the control arm. Single-arm Phase I and/or II trials that administered ICIs were also included. The primary outcomes were limited to overall survival (OS), progression-free survival (PFS), disease-free survival (DFS), and event-free survival (EFS).

### 3.2. Race- and Sex-Based Characterization

**Figure 2:**
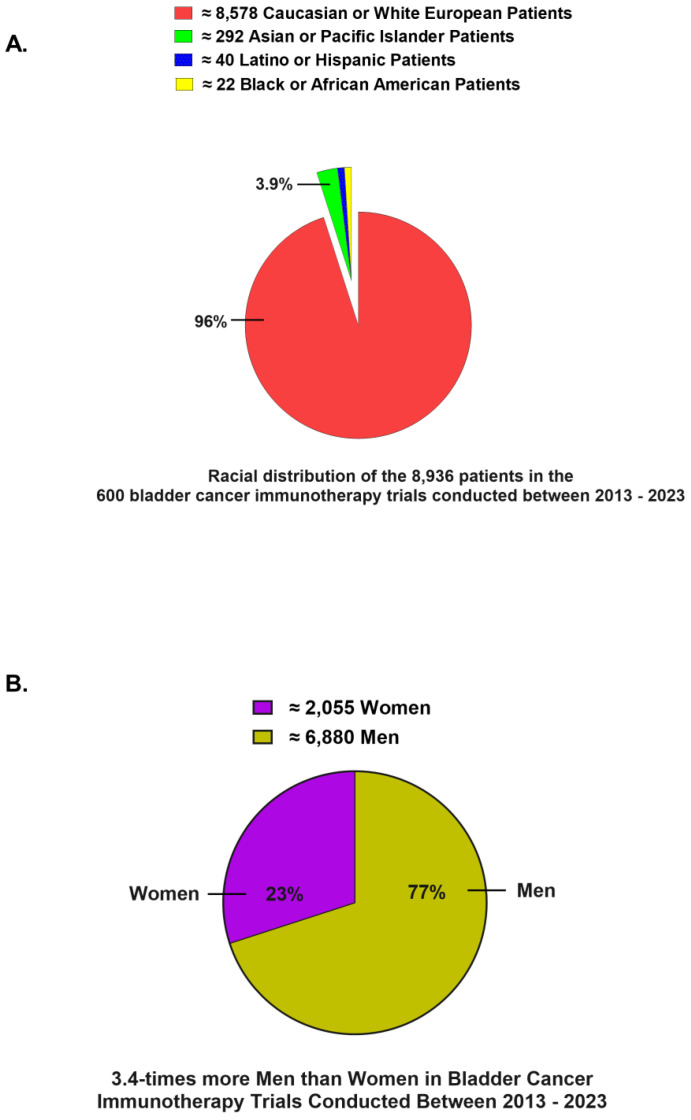
Sex and racial distribution of patients. A pie chart showing the racial (**A**) and sex (**B**) distribution of patients that received immune checkpoint inhibitors.

The recruitment of diverse subjects in clinical trials is essential for ensuring that research findings are generalizable to a broad patient population. Our analysis identified a total of 8936 patients across 109 global trials conducted between 2013 and 2023 (Figure 2A). Of these, 96 percent identified as Caucasian or White European, while less than 1% identified as Latino or BAA (Figure 2A). Furthermore, upon quantifying participants based on sex, we discovered that women accounted for 23 percent of the total patients (Figure 2B).

### 3.3. Age and Comorbidities

**Figure 3:**
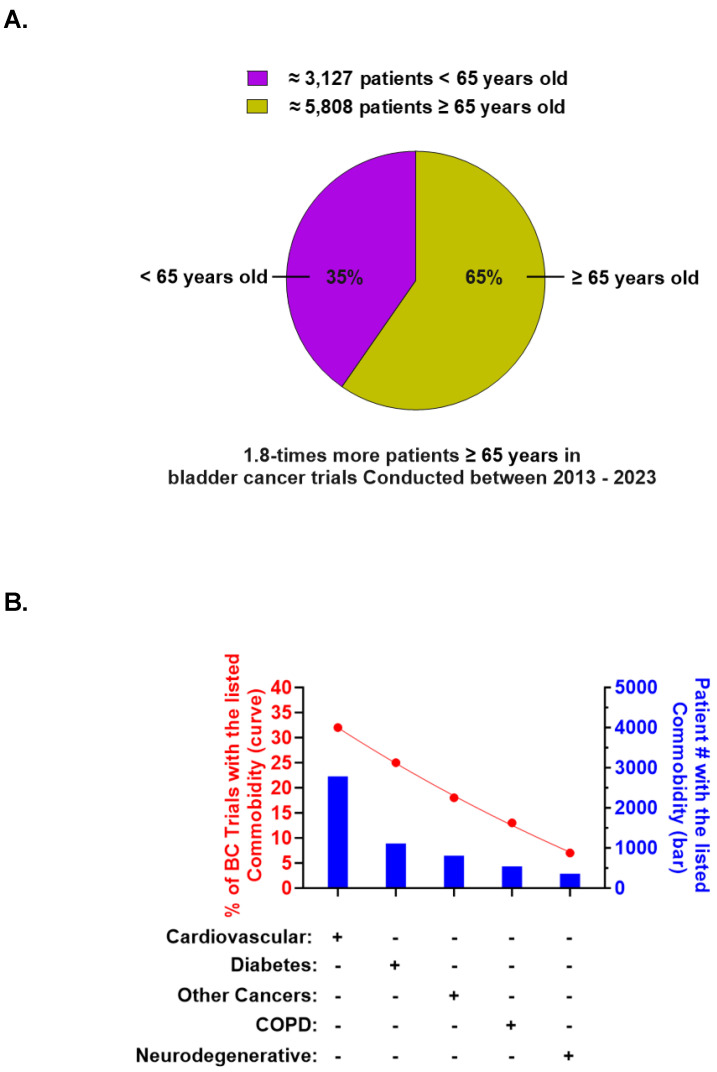
Age and comorbidities recorded in eligible trials. Graphs representing patient age (**A**) and disease comorbidities (**B**) reported in the 109 eligible studies.

We explored the contribution of older participants to the patient pool. To do so, we stratified the participants into two age groups: patients younger than 65 years and patients 65 years or older. Our analysis revealed that 65 percent of the enrolled patients were aged 65 years or older (Figure 3A). Subsequently, we analyzed our dataset for the top five comorbidities occurring alongside BC. Cardiovascular disease emerged as the leading comorbidity, which was recorded in one-third of the number of trials and approximately 30 percent of the patients (Figure 3B). Other comorbidities included diabetes, other cancer types, COPD, and cognitive disorders, ordered from most to least documented (Figure 3B).

## 4. Discussion

The goal of this systematic review was to understand demographic representation in BC clinical trials that administered ICIs to patients between 2013 and 2023. Similar studies have been conducted for BC trials administering Bacillus Calmette-Guérin (BCG) and/or chemotherapy [15,16]. ICIs have emerged as a transformative class of therapies that harnesses the body’s immune system to target and destroy cancer cells, including those in bladder tumors. Based on time of administration, ICIs can be categorized into neoadjuvant (pre-surgery) and adjuvant (post-surgery) settings. The use of neoadjuvant regimens aims to reduce tumor size before surgical intervention, while adjuvant administration seeks to eliminate residual cancer cells. Under-representation in clinical trials means that the risk and benefits of neo/adjuvant ICIs may not be fully understood across diverse populations.

Under-representation can skew results from efficacy and safety data, potentially resulting in adverse effects or reduced effectiveness for under-represented groups. For instance, studies have shown that certain antihypertensive drugs like ACE [17] inhibitors may be less effective in African American populations compared to other ethnicities. Such relevant data may only be obtained from an inclusive population. Moreover, genetic variations influencing drug metabolism can lead to different safety profiles across populations, underscoring the need for inclusive trials.

This study revealed three key findings. First, most patients enrolled in ICI trials for BC were Caucasian and approximately a quarter of the patients were female. Second, enrolled patients tended to be older, with over 65% of patients being over 65 years of age. Third, we found that comorbidities are common in BC studies, as 30% of the clinical trials listed similar comorbid conditions that affected 2800 patients.

Race- and sex-based responses to ICIs are understudied areas of ICI research but are of critical clinical interest [3,18]. Currently, a subgroup analysis in BC trials suggests that men may exhibit better responses to ICIs compared to women [11,12]. These responses have been attributed to biological differences in T-cell activation, hormonal factors, or tumor niches. However, as noted above, BC affects more men than women, and BC trials enroll a greater number of men than women, which may influence attainment of statistical significances in subgroup analyses [5,18]. Hence, sex-based comparisons may be difficult to evaluate without improving the recruitment of female participants.

Most patients in our pool of studies were Caucasian. In real-world settings, minorities have been described as having a lower incidence of and higher mortality rates related to BC [19]. Hence, understanding the reasons for the lack of diversity in patient composition within BC trials may allow for the improvement of inclusion strategies that broaden representation and improve generalizability across all populations. Recently, a few clinical trials have started to include minority recruitment strategies that may contribute to patient diversity [2,20]. Other strategies may include expanding eligibility criteria and employing targeted outreach efforts to ensure broader representation across different ethnic and racial groups. Studies have shown that BAA and Latino minorities are diagnosed with BC at a younger age and often present with more advanced disease due to socioeconomic factors [4]. Our systematic review suggests that implementing targeted outreach programs and partnerships with community organizations may improve minority recruitment plans, helping to effectively engage and enroll under-represented minority groups in clinical trials.

Additionally, we also found that enrolled patients tended to be older and that 65% of patients were older than 65 years of age. For older adult patients, ICIs may be better tolerated than traditional chemotherapy regimens [21,22]. Yet, the immune system of older adults undergoes various changes in a process known as immunosenescence [23]. These changes may impair responses to ICIs during BC treatment [24]. Similarly, clinical studies have shown that the response to ICIs in older patients may differ from that of younger patients [21]. Our analysis showed that many older patients aged 65 years and older were captured in the assessed pool of studies, indicating the importance of better understanding this population’s response to ICI treatment. Van der Heijden et al., 2023, showed that older patients greater than 65 years may exhibit poorer responses to ICIs [7]. Furthermore, older patients show selective side effects after ICI treatment [21]. These studies, coupled with our systematic review, underscore the urgent need for further research focused on the elderly as a minority cohort.

During the progression and or treatment of BC, age-related disease may present as a comorbidity [13]. Age-related changes in organ function, such as reduced renal and hepatic clearance, can affect ICI pharmacokinetics during treatment [22,24]. This can impact treatment decisions, dosing regimens, and the overall management of adverse events that result from ICI administration. Within our pool of studies, many patients had comorbidities, and as many as one in three exhibited cardiovascular disease, COPD, or diabetes (Figure 3). Some studies suggest a non-positive benefit–risk profile for BC patients with diabetes treated with ICIs. Tumor response to ICI treatment is said to vary depending on a variety of factors, including age, race, and ethnicity. However, the effect of commodities on the response to ICI treatment has yet to be assessed in patients.

ICIs used to treat various cancers can have multiple effects on the comorbidities observed in cancer patients. ICIs can also induce immune-related adverse events that affect other organs and pre-existing conditions [25]. Further, patients with autoimmune diseases may experience exacerbations of their conditions, including flare-ups of rheumatoid arthritis or lupus, due to heightened immune activity [26]. Additionally, ICIs may lead to endocrinopathies, such as thyroiditis or adrenal insufficiency, complicating the management of endocrine disorders [25]. Cardiovascular comorbidities may also be impacted, as ICI treatment can induce myocarditis or vasculitis, potentially exacerbating heart disease [27]. Furthermore, patients with pre-existing pulmonary conditions could face increased risks of pneumonitis [28].

These findings highlight the necessity of understanding the demographic representation in clinical trials. Race and ethnicity can influence BC treatment response. Race-based effects may be due to genetic, environmental, and socioeconomic factors. For example, genetic variation observed in albumin [24] levels can affect how a BAA patient metabolizes an ICI, while social determinants of health, such as access to healthcare and socioeconomic status, can impact tumor sizes at the time and age of diagnosis [16]. By understanding diverse populations in clinical trials, researchers may be able to better design and conduct more generalizable clinical trials.

Socioeconomic barriers and systemic biases significantly impact minority participation in bladder cancer clinical trials. Limited access to information due to language barriers and insufficient outreach efforts in diverse communities hinders participation. Certain eligibility criteria, such as prostate-specific antigens, can often exclude minority groups. Recruitment strategies occurring in urban areas may not be able to access rural regions. Trial staff and principal investigators also impact the ability to engage and recruit minority participants. Establishing satellite trial sites in these regions or forming partnerships with local research organizations to recruit patients in deliberate communities or specific geographic regions of interest could be considered. Even expanding satellite sites on a global scale may improve collaborations with healthcare institutions across Asia and Latin America.

## 5. Limitations

This work has several limitations. The heterogeneity among the ICI regimens that were used may impact findings. The robustness of our study is also limited by publications which may have reported duplicated data from overlapping patient populations. Several studies did not report race or ethnicity data comprehensively, limiting the ability to perform race-based analyses. The adjuvant and neoadjuvant approach of administering ICIs with chemo +/− BCG +/− surgery also may confound results. Another limitation includes the lack of more granular data regarding the clinical comorbidities, such as frailty in older patients participating in the trials. Lastly, the heterogeneity in age intervals and dissimilar endpoints may confound data synthesis. Our results are also limited by the small size of our studies, which include small numbers of minority patients.

## 6. Conclusions

Minority groups may be under-represented in most clinical trials administering ICIs. Older patients greater than 65 years often have a form of BC that presents with other comorbid conditions that may affect response to ICI treatment. In future studies, BC trials can consider these factors when designing studies.

## Data Availability

All data used for the study have been included in the manuscript.

## Abbreviations

BC	Bladder Cancer
BAA	Black or African American
BCG	Bacillus Calmette-Guérin
CT	Clinical trial
COPD	Chronic Obstructive Pulmonary Disease
EFS	Event-free survival
DFS	Disease-free survival
ICI	Immune checkpoint inhibitor
RCT	Randomized clinical trial
MIBC	Muscle-invasive Bladder Cancer
NMIBC	non-Muscle-invasive Bladder Cancer
NOS	Newcastle–Ottawa Scale
OS	Overall Survival
PFS	Progression-free survival
